# GALAD Score Detects Early-Stage Hepatocellular Carcinoma in a European Cohort of Chronic Hepatitis B and C Patients

**DOI:** 10.3390/ph14080735

**Published:** 2021-07-27

**Authors:** Clemens Schotten, Bastian Ostertag, Jan-Peter Sowa, Paul Manka, Lars P. Bechmann, Gudrun Hilgard, Claudio Marquardt, Marc Wichert, Hidenori Toyoda, Christian M. Lange, Ali Canbay, Philip Johnson, Heiner Wedemeyer, Jan Best

**Affiliations:** 1 Department of Gastroenterology and Hepatology, University Hospital Essen, 45147 Essen, Germany; clemens.schotten@uk-essen.de (C.S.); Bastian.Ostertag@uk-essen.de (B.O.); gudrun.hilgard@uk-essen.de (G.H.); marquardt.claudio@gmail.com (C.M.); christian.lange@uk-essen.de (C.M.L.); gastroenterologie@mh-hannover.de (H.W.); 2 Department of Internal Medicine, University Hospital Knappschaftskrankenhaus Bochum, Ruhr-University Bochum, 44892 Bochum, Germany; jan.sowa@rub.de (J.-P.S.); paul.manka@rub.de (P.M.); lars.bechmann@rub.de (L.P.B.); ali.canbay@rub.de (A.C.); 3 Central Laboratory, University Hospital Essen, University Duisburg-Essen, 45147 Essen, Germany; marc.wichert@uk-essen.de; 4 Department of Gastroenterology, Ogaki Municipal Hospital, Ogaki 503-8502, Japan; hmtoyoda@spice.ocn.ne.jp; 5 Department of Molecular and Clinical Cancer Medicine, University of Liverpool, Liverpool L69 3BX, UK; Philip.Johnson@liverpool.ac.uk; 6 Department of Gastroenterology, Hepatology and Endocrinology, Hannover Medical School, 30625 Hannover, Germany; 7 Department of Gastroenterology, Hepatology and Infectious Diseases, Otto-von-Guericke University Magdeburg, 39120 Magdeburg, Germany

**Keywords:** hepatocellular carcinoma, HCC, chronic hepatitis B, chronic hepatitis C, HBV, HCV, AFP, AFP-L3, DCP, GALAD

## Abstract

Despite vaccination programs and direct antiviral treatments, the incidence of virus-related hepatocellular carcinoma (HCC) remains high, while ultrasound-based detection rates for early-stage HCC is continuously low. To address this insufficiency, we set out to characterize whether the GALAD score, which incorporates gender, age, and serum levels of AFP, AFP isoform L3 (AFP-L3), and des-gamma-carboxy-prothrombin (DCP), can improve early-stage HCC detection in a Caucasian HBV/HCV cohort. In a retrospective German single-center study, 182 patients with HBV, 223 with HCV and 168 with other etiology (OE) of chronic liver disease (CLD) were enrolled. HCC was confirmed in 52 HBV, 84 HCV and 60 OE CLD patients. The diagnostic performance of the single biomarkers in HCC detection was compared to the GALAD model. At initial diagnosis, most patients were at (very) early BCLC 0 (*n* = 14/7%) or A (*n* = 56/29%) or intermediate stage BCLC B (*n* = 93/47%) HCC in all three subgroups. In the BCLC 0/A cohort, GALAD exhibited an AUC of 0.94 discriminating HCC from non-HCC, surpassing AFP (AUC 0.86), AFP-L3 (AUC 0.83) and DCP (AUC 0.83). In the HBV population, GALAD achieved an AUC of 0.96, in HCV an AUC of 0.98 and in OE an AUC of 0.99, clearly superior to the biomarkers alone. Furthermore, in HCV patients GALAD showed a significantly higher specificity (89%) versus AFP (64%) alone. In chronic viral hepatitis, the GALAD model showed superior performance in detection of early-stage HCC, while exhibiting higher specificity in HCV patients compared to AFP alone. We conclude that the GALAD score shows potential for HCC surveillance in Caucasian HBV/HCV patients.

## 1. Introduction

The worldwide annual incidence of hepatocellular carcinoma (HCC) almost parallels its mortality rate being the sixth most frequent malignancy and the second most common cause of cancer-related death [1]. The global incidence of HCC shows a continuous increase since the 1980s [2]. Despite Hepatitis B (HBV) vaccination programs and effective direct antiviral agents (DAA) for treatment of chronic hepatitis C (HCV), the incidence of virus-related HCC remains high. HCV eradication by antiviral treatment reduces but does not eliminate HCC risk. Patients with HCV-related cirrhosis require HCC surveillance even after sustained virologic response (SVR) due to a persistent risk of HCC even years after SVR [3].

Despite limited performance in patients who are obese or have small tumors, HCC surveillance is routinely based on ultrasound and only a few guidelines recommend additional determination of serum alpha-fetoprotein (AFP) levels [4,5,6]. Recent data from large scale retrospective trials clearly indicate that complimentary AFP measurement in addition to ultrasound markedly increases detection rate of early-stage HCC [7]. Furthermore, an AFP based model that was recently developed in a Japanese longitudinal patient collective could facilitate stratification of patients to HCC high-risk versus low-risk groups and could be used to select patients for surveillance in the near future [8]. According to European experiences, a large proportion of early-stage HCCs are AFP negative, mirroring its limited performance in early-stage detection [9]. To address this diagnostic problem, it has been discussed to lower the AFP cutoff levels; however, slight AFP elevations frequently occur during the course of chronic viral hepatitis and in the absence of HCC, resulting in potentially false-positive results [10,11].

Consequentially, additional biomarkers to AFP are urgently required to close the diagnostic gap. Previous studies indicate that AFP isoform L3 (AFP-L3), and des-gamma-carboxy prothrombin (DCP) are independent but complementary markers for HCC detection [12,13,14]. Combination of these three aforementioned biomarkers demonstrated a superior detection of HCC with no significant decrease in specificity [15,16]. For further improvement, the GALAD score was developed, including two independent HCC risk factors: patient gender (G) and age (A). The combination of these two demographic factors with AFP-L3 (L), AFP (A) and DCP (D) facilitates a detection of early-stage HCC with a sensitivity of 86 % and specificity of 90% in a British cohort [17]. These results were confirmed in large scale international multicenter studies of different etiologies for HCC [18,19].

Despite the broad clinical evidence of the GALAD models superiority for HCC detection in Asian HBV and HCV cohorts, its application in Caucasian populations remains low. Therefore, the current study aims to (1) investigate the diagnostic efficacy of the biomarkers AFP, AFP-L3 and DCP for HCC detection, either alone, in combination or as part of the GALAD score, and (2) determine whether the GALAD score exhibits superior specificity compared to AFP, that frequently generates false-positive results in correlation to the virus related necroinflammation.

## 2. Results

### 2.1. Demography

A total of 573 patients with chronic liver disease were included in this German single-center cohort trial. Demographic and clinical data of the study population are displayed in Table 1. The study population was divided into three subgroups: The first cohort included 182 HBV patients (52 with HBV-HCC), the second cohort 223 HCV patients (84 with HCV-HCC) and the third 168 patients with liver disease of other etiology (OE) (60 OE-HCCs). (Very) early-stage HCC (BCLC 0/A) was found in 70 patients (36%), whereas intermediate to advanced stage HCC (BCLC B/C) was prevalent in 126 (64%) patients (Table 1). BCLC-HCC stage in HCV patients was significantly less advanced as compared to HBV and OE (*p* ≤ 0.05 by Kruskal Wallis Test).

Prevalence of type II diabetes and history of tobacco use were significantly more frequent in HCC patients, independent of etiology of liver disease, age, and cirrhosis. Complications of liver cirrhosis, such as portal hypertension and hepatic encephalopathy, were observed at a higher frequency in HCC patients (*p*-value ≤ 0.05) (Table S1, Supplementary Materials). Relevant comedications for treatment of metabolic, cardiovascular, hepatic, and other diseases are depicted in Table S2.

### 2.2. Performance of GALAD Compared to Individual Markers in All Etiologies

**Figure 1:**
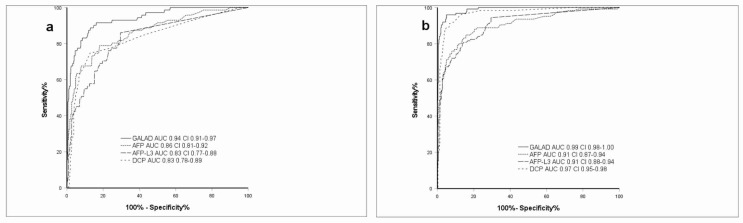
GALAD in early-HCC and intermediate to advanced HCC. (**a**) ROC-Curve for GALAD, AFP, AFP-L3, DCP, GALAD in early HCC (BCLC 0/A); (**b**) ROC-Curve for GALAD, AFP, AFP-L3, DCP in intermediate to advanced stage HCC (BCLC B/C/D); DeLong test *p* ≤ 0.001. GALAD: gender, age, AFP-L3, AFP, DCP score, HCC: hepatocellular carcinoma, HBV: chronic hepatitis B infection, HCV: chronic hepatitis C infection, AFP: alpha-fetoprotein, AFP-L3: ratio of LCA-reactive AFP to total AFP, DCP: des-gamma-carboxyprothrombin, AUC: area under the curve, CI: confidence interval.

For BCLC 0/A patients the GALAD model exhibited a superior AUC of 0.94 compared to AFP, AFP-L3 and DCP AUCs ranging between 0.83–0.86 (all *p* ≤ 0.05). In BCLC B/C patients, GALAD featured an AUC of 0.99 with AFP, AFP-L3 and DCP ranging from 0.91 to 0.97 (all *p* ≤ 0.05) (Figure 1).

### 2.3. Demographics and GALAD Performance in Chronic HBV

HBV patients without HCC showed significantly lower prevalence of cirrhosis compared to HBV-HCC patients (HCC 69.2%, non-HCC 12.3%; *p* ≤ 0.05). HCC patients were predominantly at Child Pugh stage A (HCC 48.1%, non-HCC 10%; *p* = 0.6). Many patients with HCC were BCLC stage 0/A (34.6%) or stage B (50%). HCCs of 13.5% and 1.9% were classified as BCLC stage C or D (Table 1).

**Figure 2:**
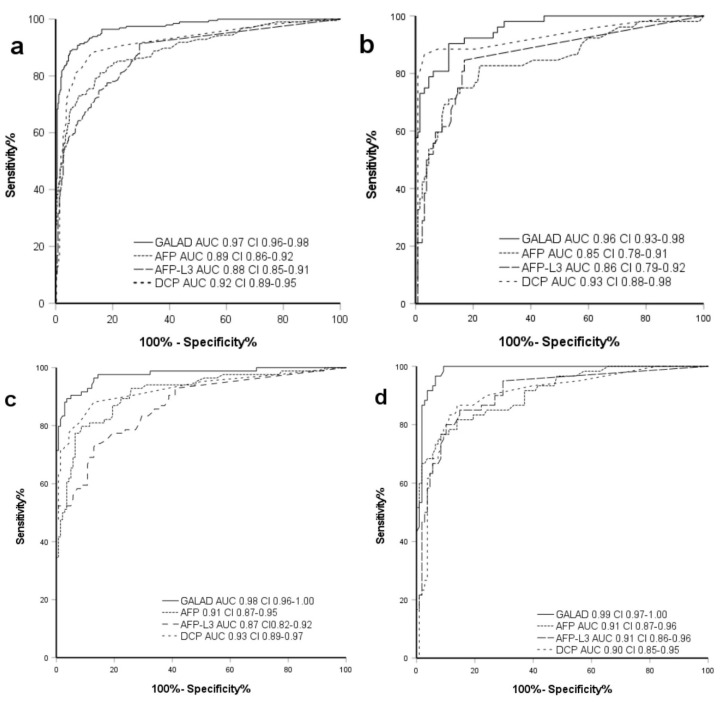
ROC-curve for GALAD vs. individual biomarkers in all etiologies. ROC-Curve for GALAD, AFP, AFP-L3 and DCP for HCC detection; (**a**) all patients, (**b**) HBV, (**c**) HCV, (**d**) others. DeLong test *p*-Value: All *p*-values ≤ 0.001. HCC: hepatocellular carcinoma, HBV: chronic hepatitis B infection, HCV: chronic hepatitis C infection, AFP: alpha-fetoprotein, AFP-L3: ratio of LCA-reactive AFP to total AFP, DCP: des-gamma-carboxyprothrombin, AUC: area under the curve, CI: confidence interval.

HDV coinfection was detected in seven HCC (13.5%) and nine non-HCC patients (6.9%). Mean values of HBV-DNA at first diagnosis or first presentation in our hospital or outpatient clinic in non-HCC patients was 2092 IU/mL, which is close to the EASL guideline threshold for initiation of HBV specific treatment (Table S3). Longitudinal evaluation proved no relevant viral load (0 IU/mL (IQR 61)) in both cohorts. Non-HCC patients exhibited a predominant ALT elevation (64.5 U/L HCC, 60.7 U/L non-HCC; *p* = 0.33) with a De Ritis ratio under 0.8, which is pathognomonic for hepatitic liver injury. HCC patients showed a leading AST elevation (71.8 U/L, 46.7 U/L HCC, non-HCC; *p* ≤ 0.05) resulting in a De Ritis ratio of 1.1 (*p* ≤ 0.05) (Table 2). In 46 (35.4%) non-HCC patients, no HBV specific treatment was performed compared to five (9.6%) of the HCC patients at their first diagnosis (*p* ≤ 0.05). Common treatments were tenofovir (38.5% HCC, 27.7% non-HCC; *p* = 0.19) and entecavir (50% HCC, 36.9% non-HCC; *p* = 0.33). Other treatments (15.4% HCC, 11% non-HCC; *p* = 0.25) included lamivudine, adefovir, and telbivudine (Table S3). Liver synthesis function as depicted in Table 1 and Table 2 indicates a more advanced liver disease stage in HBV-HCC patients with higher levels of bilirubin and lower albumin levels, and subsequently worse ALBI scores compared to the non-HCC cohort (for each value *p* ≤ 0.05). HCC-specific biomarker levels were significantly higher in HBV-HCC patients (Table 3). With standard cutoff-levels applied for AFP (>20 ng/mL) and GALAD (>−0.63), AFP featured a sensitivity of 48.1% versus 76.9% for GALAD, with similar values for specificity (both 95.4%). In 16 (31%) of the AFP negative patients, HCC could be detected by GALAD. Three (2%) patients were false-positive by GALAD while being negative for AFP. In addition, one AFP positive HCC patient (2%) was not detected by GALAD. In the HBV cohort, GALAD achieved an AUC in the ROC-analysis of 0.96, which is superior to each biomarker applied individually (AUC ranging between 0.85–0.93) (Figure 2).

### 2.4. Demographics and GALAD Performance in Chronic HCV

In the chronic HCV cohort, the majority of patients were genotype (GT) I (57.1% HCC, 66.9% non-HCC), with GT III being less common (16.7 HCC; 22.3% non-HCC). Antiviral treatment encompassed DAAs (56.0% HCC, 92.8% non-HCC) and interferon-based regimens (35.7% HCC, 20.1% non-HCC). A total of 46 (55.4%) HCC patients and 126 (90.6%) of the non-HCC patients achieved SVR under antiviral treatment (Table S4, Supplementary Materials).

Tumor stages, according to BCLC, were (very) early HCCs in 50% of the cases, and intermediate stage was prevalent in 32% of the cases. Tumor extension was limited multifocal (1.93 (SD 1.11)) and under the margin of 5 cm as shown in Table 1 in the Milan classification [20] for unifocal tumors.

In non-HCC patients, a prevalence of type II diabetes was significantly less common (28.6% HCC; 14.4% non-HCC; *p* ≤ 0.05), while exhibiting significantly higher BMI compared to the HCC cohort (27.9 kg/m² HCC, 25.2 kg/m² non-HCC; *p* ≤ 0.05). Cirrhosis was evident in 91% of HCC patients and trending to a higher Child Pugh Score compared to non-HCC patients (41% cirrhosis) (Table 1). Higher levels of bilirubin and lower levels of albumin were found in the HCC group (*p* ≤ 0.05) (Table 2). At standard cutoff for AFP (>20 ng/mL) and GALAD (>−0.63), 22 AFP negative HCC patients (26%) were additionally detected by GALAD. Though, 3 (2%) patients were GALAD positive while being AFP negative without HCC evidence. One (1%) AFP positive HCC patient was not detected by GALAD. GALAD exhibited a sensitivity of 89.3% in contrast to AFP with 64.3% with a similar specificity (95.7%/95% GALAD/AFP) (Tables S5 and S6). The AUC for GALAD was significantly higher with 0.98 compared to each biomarker applied individually (ranging between 0.87–0.93; *p* ≤ 0.05) (Figure 2).

### 2.5. Demographics and GALAD Performance in Other Etiologies of Chronic Liver Disease

The cohort with other etiologies (OE) contains patients with non-alcoholic fatty liver disease (NAFLD), alcoholic liver disease and with autoimmune hepatitis, primary sclerosing cholangitis or primary biliary cholangitis. Compared to the patients with chronic viral hepatitis, in the OE cohort the HCC patients exhibited higher age and a significantly higher rate of metabolic comorbidities like type II diabetes, arterial hypertension and obesity (all *p* ≤ 0.05). Tumor stages were evenly distributed around the intermediate stage (66.7%) HCC with limited multifocality and a mean size of the major nodule of 6.64 (SD 4.05) cm as shown in Table 1. OE HCC patients also showed a higher rate of underlying cirrhosis (81% HCC, 45% non-HCC) with a tendency to a worse liver synthesis function. Serological biomarkers as well as the GALAD score were significantly elevated in the HCC group (*p* ≤ 0.05). The GALAD score correctly detected HCC in 18 (80%) of the AFP negative patients, while 6 (6%) AFP negative non-HCC patients were false-positive by GALAD. ROC analysis showed an AUC of 0.99 for GALAD and 0.91 for AFP (*p* ≤ 0.05). Sensitivity and specificity were 89.3% and 95.7% for the GALAD score, in the non-HCC group they were 64.3% and 95%, respectively.

## 3. Discussion

Our analysis validates the utility of the GALAD model in a Caucasian cohort of chronic hepatitis B and C patients in absence or presence of cirrhosis for detection of early-stage HCC. GALAD demonstrated a clear superiority in detection of (very) early-stage BCLC stage 0/A HCC compared to the biomarkers AFP, AFP-L3 and DCP alone. In the HCV population, where AFP levels show relevant fluctuations due to hepatic necroinflammation, GALAD exhibited a significantly higher specificity in discriminating HCC from non-HCC.

In the intermediate to advanced HCC BCLC stages B, C and D the GALAD score was able to discriminate HCC from non-HCC over all etiologies, with an AUC of 0.99, compared to AFP alone with an AUC of 0.91. It is important to note that our study included a large proportion of HCC early-stage patients at BCLC stage 0/A (36%), representative of the population that could undergo curative treatment. In this subgroup the GALAD exhibited an even better performance compared to AFP with an AUC of 0.94 versus 0.86 for AFP alone. When patients with HCC due to HBV were analyzed separately the GALAD achieved an AUC of 0.96 versus AFP with an AUC of 0.85. When the analysis was limited to patients with HCV, AUCs of 0.98 for the GALAD and of 0.91 for AFP were reached. This data demonstrates a clear superiority in discriminating HCC from non-HCC, as well as in patients with viral hepatitis for GALAD.

One reason for the better performance of GALAD compared to AFP, in their ability to discriminate HCC from non-HCC, may be that AFP serum concentrations are affected by hepatitis virus-induced liver inflammation. Elevated serum AFP-levels > 10 ng/mL (>20 ng/mL) in non-HCC patients were documented most frequently in HCV patients with 11.5% (5.0%) and in HBV patients with 8.5% (4.6%), compared to the cohort with liver diseases of other etiology with 7.4% (1.9%). Those findings are consistent with current literature and are presumably correlated to virus-related hepatic necroinflammation [21], leading to potentially unnecessary cross-sectional imaging procedures, required to exclude underlying HCC. Considering the results presented here, the GALAD would probably not only detect HCC with higher sensitivity but also lead to higher specificity in settings with chronic hepatitis surveillance.

The two main problems regarding surveillance or screening for HCC in chronic viral hepatitis are non-cirrhotic HCC in patients with HBV, and SVR in patients of HCV. During long-term chronic HBV-infection, HCCs can frequently arise even in the absence of underlying cirrhosis. In the real-world experience, the non-cirrhotic HBV population that is frequently “under-screened”, is diagnosed at more advanced HCC stages, making them ineligible for curatively intended treatment approaches. In parallel, since DAA treatment for chronic HCV was introduced in 2014, most patients achieve SVR. While HCV eradication by antiviral treatment does reduce HCC risk, the predisposing cirrhosis persists. HCV patients with confirmed cirrhosis require HCC surveillance independent of their SVR status since elevated HCC risk remains even many years after SVR. For those aforementioned at-risk populations, international HCC-guidelines recommend six monthly ultrasound surveillance intervals, either with or without additional determination of AFP-levels. However, hepatic parenchymal inflammation in the context of chronic HCV can cause an increase in AFP with false-positive screening results for HCC [22]. Most early stage HCCs are AFP negative in European studies, making this marker alone inadequate for early tumor recognition [9]. Consequently, AFP determination for HCC surveillance has been disapproved by US-American and European guidelines. Furthermore, the prevalence of concomitant hepatic steatosis for chronic HCV ranges from 40% to 86% (mean ~55%) in western countries, thus occurring more frequently than in the general population [23]. In steatotic livers, sensitivity of current ultrasound-based approaches to detect early-stage HCC is insufficient [24,25]. Thus, for surveillance of patients with chronic viral hepatitis and a specific risk profile like non-cirrhotic, steatotic HBV or HCV with SVR after DAA-treatment, ultrasound-based approaches and AFP alone are insufficient regarding sensitivity and should be complemented or even replaced by the GALAD measurement.

A large US-multicenter trial conducted by Tzartzeva et al. comprising 13.367 patients demonstrated that the poor sensitivity of ultrasound alone (47%) in detection of early-stage HCC could be significantly enhanced by additional AFP determination (63%) [7]. However, this sensitivity is still ineligible for clinical application. To further improve diagnostic efficacy, the GALAD model was developed and includes sex, age, AFP, AFP-L3 and DCP, which combined are superior in HCC detection in Asian patient cohorts [15,16]. The GALAD model exhibited excellent performance in HCC detection in multiple validation studies, including very large cohorts and early-stage HCC (BCLC 0/A) of various etiologies [18,26,27].

A limitation of this German monocentric study is its retrospective nature; however, we were able to demonstrate that GALAD outperforms the application of AFP alone for HCC detection. For future prospective studies, a comparison to ultrasound vs. GALAD determination, or combination of both, is mandatory. Another limitation might be the lack of comparison to other scoring systems as ALBI or PALBI [28].

## 4. Materials and Methods

### 4.1. Ethics

The study was approved by the institutional review board/ethics committee (reference number 21-9828-BO, at the meeting held on 29 March 2021) of the University Hospital Essen and carried out in accordance with the Declaration of Helsinki in its latest revision (2013 Fortaleza, Brazil). This research was a retrospective study approved by the ethics committee of the University Hospital and the requirement of informed consent was waived.

### 4.2. Retrospective Cohort Study

The performance of the GALAD Model was evaluated in a single center case control dataset at the University Hospital Essen in Germany. A total of 573 patients with liver disease of different etiology were enrolled between 2008 and 2020. A total of 196 patients in this cohort had HCC, either in the context of chronic viral hepatitis B or C (136 patients), or of other non-viral origins (60 patients).

HBV infection was defined by either seropositivity for the hepatitis B surface antigen (HBsAg) and/or HBV-DNA. Patients with the Hepatitis-Delta (HDV) coinfection were included. In compliance with European Association for the Study of the Liver (EASL) guidelines, patients with HBe-Ag positivity or chronic HBV with evidence of HBV-DNA of more than 2.000 IU/mL and/or evidence of inflammation, fibrosis or cirrhosis were treated with nucleoside/nucleotide analogues [29].

History of HCV was defined by detection of Anti-HCV and/or HCV-RNA. Patients were treated in accordance with EASL guidelines. Treatment was carried out with combinations of peginterferon and/or ribavirin prior to approval of DAA agents. Since the availability of DAA treatments, interferon-free treatments became a standard of care for patients with and without cirrhosis. DAA regime selection was performed according to the current guidelines and drug availability [30].

Liver cirrhosis was diagnosed by either assessment of histology, or typical imaging in combination with overt clinical evidence of hepatic encephalopathy or portal hypertension in the context of chronic liver disease such as splenomegaly, esophageal varices, or ascites. Severity of cirrhosis was categorized in clinical routine by a Child-Pugh-score.

Patients at risk for HCC were enrolled into surveillance programs according to German HCC guidelines. An ultrasound was performed in the absence or presence of additional AFP determination every six months [4].

HCC was confirmed according to EASL guidelines via histology or in case of cirrhosis by at least one positive imaging modality (dynamic contrast enhanced computed tomography, magnetic resonance imaging or contrast enhanced ultrasound). Tumor stage was characterized by the Barcelona Clinic Liver Cancer (BCLC) staging system [5]. Comorbidities, long-term medication, and laboratory values were assessed by institutional electronical medical health records.

The following serological biomarkers were assessed during clinical routine. Typical timepoints of biomarker assessment were prior initiation of treatment with a new antiviral drug, routine HCC screening or in the case of HCC at initial diagnosis. AFP, AFP-L3, and DCP were measured in the same serum sample (stored at −20 °C) using the μTAS Wako^TM^ i30 fully automated immunoanalyzer (FUJIFILM Wako Chemicals Europe GmbH, Neuss, Germany).

Analysis was performed using liquid-phase binding assays with subsequent capillary electrophoresis and fluorescence detection in microchips. The assay’s sensitivities were 0.3 ng/mL for AFP and 0.1 ng/mL for DCP. The percentage of AFP-L3 was determined in samples where both subfractions (AFP-L1 and AFP-L3) were >0.3 ng/mL.

The GALAD Score was calculated according to the equation (Z = −10.08 + 0.09 × age + 1.67 × gender + 2.34 × log10 (AFP) + 0.04 × AFP-L3 + 1.33 × log10 (DCP)). Gender was defined as 1 for males and 0 for females. A cutoff for AFP of ≤20 ng/mL and GALAD ≥−0.63 were defined as suspicious for HCC [17].

### 4.3. Statistical Methods

Statistical analyses were performed with SPSS 27 (IBM, Armonk, NY, USA) or Prism 9 (GraphPad, La Jolla, CA, USA). Parameters were compared between HCC and non-HCC within each cohort using student’s *t*-Test for continuous, normally distributed variables, and the Mann-Whitney-test for ordinal scaled, nonparametric variables, or chi-square tests for dichotomous variables. Dependent variables were evaluated, by either a paired student’s *t*-Test or paired Wilcoxon, as feasible.

The GALAD model and single biomarker models were compared using receiver operating characteristic (ROC) curves and the corresponding area under the curves (AUC) with the established cut-off point of −0.63. AUCs were compared according to DeLong et al. [13]. Sensitivity and specificity were calculated for the standard cutoff.

All *p*-values are two sided with a significance level of 0.05 and adjusted for multiple testing. Standard deviations or interquartile ranges were used as feasible.

## 5. Conclusions

In conclusion, we were able to demonstrate that the biomarker-based GALAD score clearly outperforms the biomarkers AFP, DCP and AFP-L3 used individually for early-stage HCC detection in a Caucasian cohort of chronic hepatitis B or C patients. GALAD showed robust performance in discrimination of HCC patients, independent of tumor burden, extent of liver disease and viral load. Further validation of GALAD’s diagnostic performance in future international multicenter prospective studies of patients suffering from viral hepatitis B or C is warranted. Additionally, a direct comparison to ultrasound examinations should be performed.

## Figures and Tables

**Table 1:** Demographics, Characteristics of HCC and non-HCC-Group for HBV, HCV and others.

Parameter	Units	HBV			HCV			OE		
		HCC (*n* = 52)	Non-HCC (*n* = 130)	*p*-Value	HCC (*n* = 84)	Non-HCC (*n* = 139)	*p*-Value	HCC (*n* = 60)	Non-HCC (*n* = 108)	*p*-Value
**Age (SD)**	(Years)	62.5 (11.8)	47.2 (13.9)	≤0.05 ^b^	64.3 (9.4)	57.3 (13.2)	≤0.05 ^b^	69.6 (7.9)	59.7 (14.5)	≤0.05 ^b^
**Sex m/f**	N	44/8	91/39	0.06 ^a^	59/25	79/60	≤0.05 ^a^	49/11	45/63	≤0.05 ^a^
**Child-Pugh grade** ***n* (%)**	No cirrhosis (n%)	16 (30.8)	114 (87.7)	≤0.05 ^a^	7 (8.3)	81 (58.3)	≤0.05 ^a^	11 (18.3)	59 (54.6)	≤0.05 ^a^
	A (n%)	25 (48.1)	13 (10.0)	0.60 ^a^	54 (64.3)	49 (35.4)	0.16 ^a^	31 (51.7)	35 (32.4)	0.50 ^a^
	B (n%)	10 (19.2)	3 (2.3)		23 (27.4)	9 (6.5)		15 (25.0)	10 (9.3)	
	C (n%)	1 (1.9)	0 (0.0)		0 (0.0)	0 (0.0)		4 (6.7)	4 (3.7)	
**MELD Score**	Mean (SD)	11.00 (3.06)	10.60 (6.47)	0.90 ^b^	12.64 (3.92)	11.20 (4.21)	0.16 ^b^	11.86 (4.06)	11.65 (4.35)	0.90 ^b^
**BMI (SD)**	(kg/m^2^)	25.91 (3.20)	26.34 (4.49)	0.59 ^b^	25.24 (4.69)	27.9 (4.99)	≤0.05 ^b^	29.53 (4.58)	27.81 (5.95)	≤0.05 ^b^
**ALBI Score (IQR)**	(−2.60)	−2.92 (0.67)	−3.08 (0.38)	≤0.05 ^c^	−2.71 (0.65)	−3.05 (0.58)	≤0.05 ^c^	−2.69 (0.73)	−3.08 (0.67)	≤0.05 ^c^
**ALBI grade, *n* (%)**	1 (n%)	32 (68.1)	113 (92.6)	0.68 ^a^	53 (63.9)	110 (82.1)	0.60 ^a^	30 (55.6)	24 (77.4)	0.84 ^a^
	2 (n%)	11 (23.4)	8 (6.6)		29 (34.9)	24 (17.9)		20 (37.0)	6 (19.4)	
	3 (n%)	4 (8.5)	1 (0.8)		1 (1.2)	0 (0.0)		4 (7.4)	1 (3.2)	
**BCLC Stage**	0 (%)	5 (9.6)			8 (9.5)			1 (1.7)		
	A (%)	13 (25.0)			34 (40.5)			9 (15.0)		
	B (%)	26 (50.0)			27 (32.1)			40 (66.7)		
	C (%)	7 (13.5)			15 (17.9)			9 (15.0)		
	D (%)	1 (1.9)			0 (0.0)			1 (1.7)		
**Nodules**	(number) (SD)	1.79 (1.07)			1.93 (1.11)			1.92 (1.10)		
**Tumor size** **(major nodule)**	(cm) (SD)	5.11 (3.28)			4.19 (2.85)			6.64 (4.05)		

*p*-Value: All *p*-Values are calculated between HCC and non-HCC within an etiology; ^a^ CHI-square, applied for two dichotomous variables; ^b^ Students *t*-Test, applied for normal distributed continuous data; ^c^ Mann-Whitney-U-Test, applied for non-parametric and non-normal distributed data; all *p*-Values are two sided with a significance level of ≤ 0.05. HCC: hepatocellular carcinoma, HBV: chronic hepatitis B infection, HCV: chronic hepatitis C infection, OE: other etiologies, f: female, m: male, MELD: Model of End-Stage liver disease, ALBI: albumin-bilirubin score, IQR: interquartile range, BCLC: barcelona clinic liver cancer, SD: standard deviation.

**Table 2:** Lab values of HCC group and control group for HBV, HCV and others.

Parameter (Normal Values)	HBV			HCV			OE		
	HCC (*n* = 52)	Non-HCC (*n* = 130)	*p*-Value	HCC (*n* = 84)	Non-HCC (*n* = 139)	*p*-Value	HCC (*n* = 60)	Non-HCC (*n* = 108)	*p*-Value
**AST (SD)** (U/L; m < 50/f < 35)	71.8 (57.87)	46.7 (105.35)	≤0.05	95.8 (91.81)	50.8 (51.01)	≤0.05	111.4 (250.43)	42.4 (25.04)	≤0.05
**ALT (SD)** (U/ML; m < 50/f < 35)	64.5 (46.63)	60.7 (138.46)	0.33	74.8 (60.05)	56.9 (67.32)	≤0.05	57.8 (45.21)	48.8 (37.85)	0.18
**De Ritis (IQR)** (0.6–0.8)	1.1 (0.6)	0.8 (0.5)	≤0.05	1.2 (0.8)	1.0 (0.6)	≤0.05	1.3 (0.2)	0.9 (0.6)	≤0.05
**GGT (SD)** (U/L; m < 55/f < 35)	237.3 (441.32)	44.8 (61.10)	≤0.05 ^a^	187.5 (194.67)	81.0 (118.50)	≤0.05 ^a^	322.6 (318.18)	152.3 (222.38)	≤0.05 ^a^
**Bilirubin (IQR)** (mg/dL; 0.3–1.2)	0.8 (0.88)	0.7 (0.40)	≤0.05 ^b^	0.9 (0.70)	0.65 (0.60)	≤0.05 ^b^	0.9 (0.80)	0.7 (0.80)	≤0.05 ^b^
**Albumin (SD)** (g/dL; 3.4–4.8)	4.0 (0.68)	4.5 (0.41)	≤0.05 ^a^	4.1 (0.56)	4.3 (0.46)	≤0.05 ^a^	3.9 (0.65)	4.3 (0.58)	≤0.05 ^a^
**Creatinin (IQR)** (mg/dL; 0.6–1.1)	0.99 (0.17)	0.93 (0.19)	0.08 ^b^	0.94 (0.24)	0.93 (0.21)	0.68 ^b^	1.02 (0.33)	0.98 (0.21)	0.32 ^b^

*p*-Value: All *p*-Values are calculated between HCC and non-HCC within an etiology; ^a^ Students *t*-Test, applied for normal distributed continuous data; ^b^ Mann-Whitney-U-Test, applied for non-parametric and non-normal distributed data; all *p*-Values are two sided with a significance level of ≤ 0.05. OE: other etiology, HCC: hepatocellular carcinoma, HBV: chronic Hepatitis B infection, HCV: chronic Hepatitis C infection, AST: aspartate aminotransferase, ALT: alanine aminotransferase, GGT: gamma glutamyl transferase, SD: standard deviation, IQR: interquartile range.

**Table 3:** HCC Serobiomarker of HCC group and non-HCC group for HBV, HCV and others.

Parameter	Units	HBV			HCV			OE		
		HCC (*n* = 52)	non-HCC (*n* = 130)	*p*-Value	HCC (*n* = 84)	non-HCC (*n* = 139)	*p*-Value	HCC (*n* = 60)	non-HCC (*n* = 108)	*p*-Value
AFP (>10 ng/mL)	n (%)	31 (59.6)	11 (8.5)	≤0.05 ^a^	67 (79.8)	16 (11.5)	≤0.05 ^a^	45 (75.0)	8 (7.4)	≤0.05 ^a^
AFP (>20 ng/mL)	n (%)	25 (48.1)	6 (4.6)	≤0.05 ^a^	54 (64.3)	7 (5.0)	≤0.05 ^a^	40 (66.7)	2 (1.9)	≤0.05 ^a^
AFP (>50 ng/mL)	n (%)	20 (38.5)	3 (2.3)	≤0.05 ^a^	41 (48.8)	3 (2.2)	≤0.05 ^a^	30 (50.0)	0 (0.0)	≤0.05 ^a^
AFP (>100 ng/mL)	n (%)	21 (38.5)	1 (0.8)	≤0.05 ^a^	35 (41.7)	1 (0.7)	≤0.05 ^a^	23 (38.3)	0 (0.0)	≤0.05 ^a^
AFP (IQR)	ng/mL (20)	22 (38.5)	2.8 (2.0)	≤0.05 ^c^	43.3 (616.5)	4.0 (3.8)	≤0.05 ^c^	50.0 (510.7)	2.7 (2.8)	≤0.05 ^c^
AFP-L3 (IQR)	% (<10)	23 (38.5)	0.3 (0.0)	≤0.05 ^c^	16.8 (47.1)	0.3 (6.1)	≤0.05 ^c^	25.4 (42.5)	0.3 (5.9)	≤0.05 ^c^
DCP (IQR)	ng/mL (7.5)	24 (38.5)	0.3 (0.1)	≤0.05 ^c^	3.2 (37.5)	0.4 (0.2)	≤0.05 ^c^	55.8 (194.3)	0.4 (0.2)	≤0.05 ^c^
GALAD (SD)	(−0.63)	25 (38.5)	−4.38 (1.96)	≤0.05 ^b^	3.66 (4.21)	−3.21 (1.52)	≤0.05 ^b^	5.51 (4.55)	−3.81 (2.15)	≤0.05 ^b^

*p*-Value: All *p*-Values are calculated between HCC and non-HCC within an etiology; ^a^ CHI-square, applied for two dichotomous variables; ^b^ Students *t*-Test, applied for normal distributed continuous data; ^c^ Mann-Whitney-U-Test, applied for non-parametric and non-normal distributed data; all *p*-Values are two sided with a significance level of ≤ 0.05. OE: other etiology, HCC: hepatocellular carcinoma, HBV: chronic hepatitis B infection, HCV: chronic hepatitis C infection, AFP: alpha-fetoprotein, AFP-L3: ratio of lens culinaris agglutin -reactive AFP to total AFP, DCP: des-gamma carboxyprothrombin, SD: standard deviation, IQR: interquartile range.

## Data Availability

Data is contained within the article and Supplementary Material.

## Supplementary Materials

The following are available online at https://www.mdpi.com/article/10.3390/ph14080735/s1, Table S1: Comorbidities of HCC and non-HCC cohorts for HBV, HCV and other etiologies, Table S2: Medication of HCC and non-HCC cohorts for HBV, HCV and other etiologies, Table S3: Characteristics of HCC and non-HCC cohorts for HBV infection; patients have been partially treated with different antiviral drugs at the same time, Table S4: Characteristics of HCC and non-HCC cohort for HCV infection, Table S5: Contingency table for HCC and non-HCC for all, HBV, HCV and others, Table S6: Overall performance of AFP and GALAD at established cut-off values in the study cohort.
