# Supplementary material for: GALAD Score Detects Early-Stage Hepatocellular Carcinoma in a European Cohort of Chronic Hepatitis B and C Patients
Source: Pharmaceuticals (Basel). 2021 Jul 27;14(8):735. doi: 10.3390/ph14080735 (PMC8401792; doi:10.3390/ph14080735)
Supplement: Supplementary file 1 [file pharmaceuticals-14-00735-s001.zip › pharmaceuticals-1266527-supplementary.pdf]

Table S1. Comorbidities of HCC and non-HCC cohorts for HBV, HCV and other etiologies.

	HBV			HCV			OE		
	HCC (n=52)	non-HCC (n=130)	*p*-Value	HCC (n=84)	non-HCC (n=139)	*p*-Value	HCC (n=60)	non-HCC (n=108)	*p*-Value
**Tobacco** n (%)	19 (36.5)	21 (16.2)	≤0.05	43 (51.2)	38 (27.3)	≤0.05	17 (28.3)	15 (13.9)	≤0.05
**Alcohol** n (%)	18 (34.6)	18 (13.8)	≤0.05	11 (13.1	30 (21.6)	0.15	10 (16.7)	17 (15.7)	1.00
**Type II Diabetes** n (%)	10 (19.2)	10 (7.7)	≤0.05	24 (28.6)	20 (14.4)	≤0.05	26 (43.3)	27 (25.0)	≤0.05
**Arterial Hypertension** n (%)	22 (42.3)	23 (17.7)	≤0.05	30 (35.7)	46 (33.1)	0.61	39 (65.0)	38 (35.2)	≤0.05
**Coronary heart disease** n (%)	6 (11.5)	7 (5.4)	0.26	10 (11.9)	8 (5.8)	0.11	11 (18.3)	6 (5.6)	≤0.05
**Other malignancy** n (%)	5 (9.6)	6 (4.6)	0.30	13 (15.5)	11 (7.9)	0.08	9 (15.0)	10 (9.3)	0.28
**Splenomegaly** n (%)	33 (63.5)	35 (26.9)	≤0.05	50 (59.5)	57 (41.0)	≤0.05	47 (78.3)	56 (51.9)	≤0.05
**Esophageal varices** n (%)	21 (40.4)	8 (6.2)	≤0.05	38 (45.2)	20 (14.4)	≤0.05	28 (46.7)	31 (28.7)	≤0.05
**Ascites** n (%)	15 (28.8)	2 (1.5)	≤0.05	38 (45.2)	8 (5.8)	≤0.05	33 (55.0)	26 (24.1)	≤0.05
**HE** n (%)	2 (3.8)	0 (0.0)	≤0.05	11 (13.1)	3 (2.2)	≤0.05	4 (6.7)	8 (7.4)	0.84

p-Value: All *p*-Values are calculated between HCC and non-HCC within an etiology by CHI²; all *p*-Values are two sided with a significance level of ≤0.05.

Abbr.: HCC (hepatocellular carcinoma), HBV (chronic Hepatitis B infection), HCV (chronic Hepatitis C infection), HE (hepatic encephalopathy).

Table S2. Medication of HCC and non-HCC cohorts for HBV, HCV and other etiologies.

	HBV			HCV			OE		
	**HCC (n=52)**	**non-HCC (n=130)**	***p*-Value**	**HCC (n=84)**	**non-HCC (n=139)**	***p*-Value**	**HCC (n=60)**	**non-HCC (n=108)**	***p*-Value**
Antidiabetics n (%)	9 (17.3)	9 (6.9)	≤0.05	19 (22.6)	14 (10.1)	≤0.05	22 (36.7)	25 (23.1)	0.06
Antihypertensive drugs n (%)	30 (57.7)	30 (23.1)	≤0.05	60 (71.4)	60 (43.2)	≤0.05	53 (88.3)	60 (55.6)	≤0.05
Steroids n (%)	2 (3.8)	5 (3.8)	1.00	3 (3.6)	5 (3.6)	0.98	9 (15.0)	27 (25.0)	0.12
Anticoagulants n (%)	14 (26.9)	10 (7.7)	≤0.05	36 (42.9)	15 (10.8)	≤0.05	33 (55.0)	23 (21.3)	≤0.05
Diuretics n (%)	25 (48.1)	12 (9.2)	≤0.05	49 (58.3)	28 (20.1)	≤0.05	48 (80.0)	46 (42.6)	≤0.05
HE prophylaxis n (%)	14 (26.9)	3 (2.3)	≤0.05	21 (25.0)	11 (7.9)	≤0.05	13 (21.7)	23 (21.3)	1.00
PPI n (%)	34 (65.4)	19 (14.6)	≤0.05	44 (52.4)	41 (29.5)	≤0.05	40 (66.7)	68 (63.0)	0.73
Vitamin D n (%)	12 (23.1)	6 (4.6)	≤0.05	12 (14.3)	9 (6.5)	≤0.05	8 (13.3)	43 (39.8)	≤0.05

p-Value: All *p*-Values are calculated between HCC and non-HCC within an etiology by CHI²-Test; all *p*-Values are two sided with a significance level of ≤0.05.

Abbr.: HCC (hepatocellular carcinoma), HBV (chronic Hepatitis B infection), HCV (chronic Hepatitis C infection), HE (hepatic encephalopathy), PPI (proton pump inhibitors).

Table S3. Characteristics of HCC and non-HCC cohorts for HBV infection; patients have been partially treated with different antiviral drugs at the same time.

	**HCC (n=52)**	**non-HCC (n=130)**	***p*-Value**
HDV coinfection n (%)	7 (13.5)	9 (6.9)	0.23[a]
HBV DNA first visit (IQR) [IU/ml]	11.0 (1000.0)	2092.0 (121 084.8)	≤0.05[c]
HBV treatment n (%)	47 (90.4)	84 (64.6)	≤0.05[a]
Entecavir n (%)	26 (50.0)	48 (36.9)	0.33[a]
Tenofovir n (%)	20 (38.5)	36 (27.7)	0.19[a]
Others n (%)	8 (15.4)	11 (8.5)	0.25[a]

Abb.: HCC (hepatocellular carcinoma), HCV (chronic Hepatitis C infection), IQR (interquartile range).
p-Value: All *p*-Values are calculated within an etiology between HCC and non-HCC; [a] CHI², [b] Students t- test, [c] Mann-Whitney-U-Test; all *p*-Values are two sided with a significance level of ≤0.05.

Table S4. Characteristics of HCC and non-HCC cohort for HCV infection.

	**HCC (n=84)**	**non-HCC (n=139)**	***p*-Value**
HCV genotype<br>Type 1(%)/ 2(%)/ 3(%)/ 4(%)/ 6(%)	48 (57.1)/ 1 (1.2)/ 14 (16.7)/ 0 (0)/ 2 (2.4)	93 (66.9)/ 4 (2.9)/ 31 (22.3)/ 9 (6.5)/ 0 (0)	0.0593 (n.s.)
HCV RNA first visit (IQR)<br>[IU/ml]	254 600.0 (1 360 500.0)	833 000.0 (1 698 475.0)	≤0.05[c]
DAA therapy<br>n (%)	47 (56.0)	129 (92.8)	≤0.05[a]
IFN therapy<br>n (%)	30 (35.7)	28 (20.1)	≤0.05[a]
SVR<br>n (%)	45 (53.6)	126 (90.6)	≤0.05[a]

Patients have been partially treated with several antiviral drugs at the same time; HCV genotype has not been examined in all patients.
p-Value: All *p*-Values are calculated within an etiology between HCC and non-HCC; [a] CHI², [c] Mann-Whitney-U-Test; all *p*-Values are two sided with a significance level of ≤0.05.
Abbr. HCC (hepatocellular carcinoma), HCV (chronic Hepatitis C infection), DAA (direct acting antivirals), IFN (Interferon), SVR (sustained virologic response), IQR (interquartile range).

Table S5. Contingency table for HCC and non-HCC for all, HBV, HCV and others.

**HCC**							
**All**				**HBV**			
	**GALAD neg.**	**GALAD pos.**	**Total**		**GALAD neg.**	**GALAD pos.**	**Total**
AFP neg.	21	56	77 (39.3%)	AFP neg.	11	16	27 (51.9%)
AFP pos.	2	117	119 (60.7%)	AFP pos.	1	24	25 (48.1%)
Total	23 (11.7%)	173 (88.3%)	p < 0.0001	Total	12 (23.1%)	40 (76.9%)	p = 0.0023
**HCV**				**OE**			
	**GALAD neg.**	**GALAD pos.**	**Total**		**GALAD neg.**	**GALAD pos.**	**Total**
AFP neg.	8	22	30 (35.7%)	AFP neg.	2	18	20 (33.3%)
AFP pos.	1	53	54 (64.3%)	AFP pos.	0	40	40 (66.7%)
Total	9 (10.7%)	75 (89.3%)	p = 0.0009	Total	2 (3.3%)	58 (96.7%)	p = 0.1073
**Non-HCC**							
**All**				**HBV**			
	**GALAD neg.**	**GALAD pos.**	**Total**		**GALAD neg.**	**GALAD pos.**	**Total**
AFP neg.	350	12	362 (96%)	AFP neg.	121	3	124 (95.4%)
AFP pos.	8	7	14 (4%)	AFP pos.	3	3	6 (4.6%)
Total	358 (95%)	19 (5%)	p < 0.0001	Total	124 (95.4%)	6 (4.6%)	p = 0.0011
**HCV**				**OE**			
	**GALAD neg.**	**GALAD pos.**	**Total**		**GALAD neg.**	**GALAD pos.**	**Total**
AFP neg.	129	3	132 (95%)	AFP neg.	100	6	106 (98.1%)
AFP pos.	4	3	7 (5%)	AFP pos.	1	1	2 (1.9%)
Total	133 (95.7)	6 (4.3%)	p = 0.0015	Total	101 (93.5%)	7 (6.5%)	p = 0.1260

Statistics: *p*-Value calculated by Fisher’s exact test.

Abbr.: HCC (hepatocellular carcinoma), HBV (chronic Hepatitis B infection), HCV (chronic Hepatitis C infection), pos. (positive), neg. (negative), AFP (Alpha-fetoprotein).

Table S6. Overall performance of AFP and GALAD at established cut-off values in the study cohort.

All					HBV				
	**Sensitivity**	**Specificity**	**PPW**	**NPW**		**Sensitivity**	**Specificity**	**PPW**	**NPW**
GALAD	88.3%	95.0%	0.901	0.940	GALAD	76.9%	95.4%	0.870	0.912
AFP	60.7%	96.0%	0.888	0.825	AFP	48.1%	95.4%	0.806	0.821
**HCV**					**OE**				
	**Sensitivity**	**Specificity**	**PPW**	**NPW**		**Sensitivity**	**Specificity**	**PPW**	**NPW**
GALAD	89.3%	95.7%	0.926	0.937	GALAD	96.7%	93.5%	0.892	0.981
AFP	64.3%	95.0%	0.885	0.815	AFP	66.7%	98.1%	0.952	0.841

Statistics: Sensitivity, Specificity, positive predictive value (PPW) and negative predictive value (NPW) were calculated using standard cutoffs for AFP 20 ng/ml and GALAD -0.63.

Abbr.: HCC (hepatocellular carcinoma), HBV (chronic Hepatitis B infection), HCV (chronic Hepatitis C infection), pos. (positive), neg. (negative), AFP (Alpha-fetoprotein).
